# Identification of Bacterial Surface Antigens by Screening Peptide Phage Libraries Using Whole Bacteria Cell-Purified Antisera

**DOI:** 10.3389/fmicb.2017.00082

**Published:** 2017-01-26

**Authors:** Yun-Fei Hu, Dun Zhao, Xing-Long Yu, Yu-Li Hu, Run-Cheng Li, Meng Ge, Tian-Qi Xu, Xiao-Bo Liu, Hua-Yuan Liao

**Affiliations:** College of Veterinary Medicine, Preventive Veterinary Medicine, Hunan Agricultural UniversityChangsha, China

**Keywords:** phage display, mimotope, homology analysis, subcellular localization, surface protein, *Erysipelothrix rhusiopthiae*

## Abstract

Bacterial surface proteins can be good vaccine candidates. In the present study, we used polyclonal antibodies purified with intact *Erysipelothrix rhusiopthiae* to screen phage-displayed random dodecapeptide and loop-constrained heptapeptide libraries, which led to the identification of mimotopes. Homology search of the mimotope sequences against *E. rhusiopthiae*-encoded ORF sequences revealed 14 new antigens that may localize on the surface of *E. rhusiopthiae*. When these putative surface proteins were used to immunize mice, 9/11 antigens induced protective immunity. Thus, we have demonstrated that a combination of using the whole bacterial cells to purify antibodies and using the phage-displayed peptide libraries to determine the antigen specificities of the antibodies can lead to the discovery of novel bacterial surface antigens. This can be a general approach for identifying surface antigens for other bacterial species.

## Introduction

Bacterial surface proteins play an essential role in bacterial interactions with the environment, including cell-cell interactions, ion and nutrient transport, cell signaling, and antibiotic resistance; they are also important for host cell infection, and specifically for defense against host responses and induction of toxicity (Niemann et al., 2004; Lebeer et al., 2010; Schneewind and Missiakas, 2012; Chagnot et al., 2013). Given their direct interaction with the host immune system, some bacterial surface proteins are candidates for vaccine development. Vaccines based on bacterial surface and secreted molecules are already commercially available, such as Infanrix (GlaxoSmithKline Biologicals S.A., Belgium), meningococcal group B vaccine (Pfizer, USA), and pneumococcal polysaccharide conjugate vaccine (Pfizer), with many others still under development (Rodríguez-Ortega et al., 2006; Jores et al., 2009; He and De Buck, 2010; Gomez et al., 2013; Sun et al., 2014).

There are currently two approaches to identifying surface proteins for vaccine development. One is reverse vaccinology, which is used *in silico* and for high-throughput screening of the entire genome of a pathogen to identify genes encoding protective antigens. This has enabled the discovery of protective antigens against *Meningococcus* (Pizza et al., 2000), group A *Streptococcus* (Mora et al., 2005), and *Streptococcus pneumoniae* (Hava and Camilli, 2002). However, this method is time consuming and requires complex genomic analyses (Jones, 2012). Alternatively, bacterial surface proteins have been identified by a proteomics approach, including those in *Mycoplasma mycoides* (Jores et al., 2009), *Mycobacterium smegmatis* (He and De Buck, 2010), and *Streptococcus suis* serotype 2 (Geng et al., 2008). But, the steps required for surface protein separation and peptide pretreatment are inconvenient and labor intensive, and the presence of cytoplasmic proteins or peptides may affect reproducibility (Emerson et al., 2008).

Phage display is a powerful molecular technique for the rapid identification of pathogen mimotopes from anti-bacterial antibodies (Pande et al., 2010); corresponding antigens can be identified by homology analysis of mimotopes covering the entire bacterial genome. However, in practice it is difficult to identify antigens based solely on mimotopes, since homology analysis of short input sequences using the Basic Local Alignment Search Tool (BLAST) can yield hundreds of homologous proteins. Surface proteins represent only a fraction of all bacterial proteins; as such, they can be identified by searching a subcellular localization database (Yu et al., 2006; Chou and Shen, 2010; Yu et al., 2010).

To demonstrate the feasibility of the phage display-subcellular localization approach for identifying protective surface proteins, we selected *Erysipelothrix rhusiopthiae* as a model pathogen. *E. rhusiopathiae* causes swine erysipelas but has also been detected in other species, including humans (Reboli and Farrar, 1989). Swine erysipelas is distributed worldwide and has economic importance (Wang et al., 2010; Zou et al., 2015). Traditional inactivated and attenuated vaccines are effective in preventing *E. rhusiopathiae* infection, but can also fail (Imada et al., 2004; Eamens et al., 2006; Bender et al., 2010); subunit vaccines based on surface-exposed proteins represent a potential alternative. However, there are major hurdles in the development of erysipelas vaccines—including a lack of knowledge regarding its pathogenesis—and only a few surface proteins have been identified in *E. rhusiopathiae*, such as surface protective antigen, *E. rhusiopathiae* surface proteins A and C, and choline-binding protein (Shimoji et al., 2003; Ogawa et al., 2011; Shi et al., 2013; Uchiyama et al., 2014). The present study was carried out in order to identify bacterial surface protective proteins by screening peptide phage libraries using whole bacterial cell-purified antisera.

## Materials and methods

### Bacterial strains and antisera

*E. rhusiopathiae* strain (ML10.1, 16sRNA GenBank: KC661000) was isolated from a pig farm in Hunan province, China. The bacteria were incubated in tryptic-soytone-broth (TSB, Sigma) medium + 10% rabbit serum at 37°C. Eight 4-week old pigs were immunized with inactivated vaccine of *E. rhusiopathiae* by intramuscularly injection (5 × 10^10^ colony forming units/pig). After 4 weeks, the pigs were challenged intramuscularly with 3 × 10^7^ colony forming units (CFU) of *E. rhusiopathiae*. Fifteen days later, auricular vein antisera samples were collected, pooled, and used for purification of antibodies against surface proteins of *E. rhusiopathiae*. Two sera from *E. rhusiopathiae* negative pigs but immunized with inactivated vaccine of *Pasteurella multocida* (gram negative bacterium) were selected as negative control. The animal experiment was performed with the approval of the Review Committee for the Use of Animal Subjects of Hunan Agricultural University and all experimental protocols were performed according to the ARRIVE guidelines of the National Institutes of Health guide for the care and use of Laboratory animals (NIH Publications No. 8023, revised 1978).

### Affinity purification of polyclonal antibodies against *E. rhusiopathiae*

Pooled antisera of *E. rhusiopathiae* were used for antibody purification. *E. rhusiopathiae* was grown overnight in TSB 10% rabbit serum. The cells were adjusted to 10^13^ CFU, and then suspended in 5 ml antisera for 10 min at ambient temperature to permit adsorption of polyclonal antibodies. Following low-speed centrifugation the pellets were resuspended and washed 3x in TBST (50 mM Tris, 150 mM NaCl, pH 7.5, containing 0.5% Tween-20), and then resuspended in 1 ml 0.2 M glycine-HCl (pH 2.2) for 10 min to dissociate the adsorbed antibodies. Following removal of the bacteria by centrifugation, the pH of the antibody-containing supernatant was neutralized with 150 μl 1 M Tris-HCl, pH 9.1. Finally, the antibodies were purified by recombinant Staphylococcus Protein A (SPA) affinity chromatography (Pierce, USA), according to the manufacturer's instructions. Specificity, purity, and concentration of the purified polyclonal antibodies were checked by ELISA kit (CIVTEST SUIS SE/MR, Hipra, Spain), SDS-PAGE, and BCA Protein Assay Kit (Pierce, USA), respectively. Negative control sera were purified as the same method.

### Phage display library and biopanning of binding peptides

The purified polyclonal antibodies were applied for biopanning of mimotopes of *E. rhusiopathiae* by using linear random library Ph.D.™-12 and loop-constrained random library Ph.D.-C7C (New England Biolabs, USA). For the first round of biopanning, a well of a 96-well ELISA plate was coated with 1 μg antibodies and blocked with 0.5% BSA for 1 h. Next, 5 μl of the library [5 × 10^10^ plaque forming unit (pfu)] with 95 μl of TBST (Tween-20, 0.1%) was added and the plate was rocked gently for 30 min at ambient temperature. Following washing 10x with TBST (Tween-20, 0.1%), any bound phage remaining were eluted with 100 μl 0.2 M glycine-HCl, pH 2.2 (eluting buffer). After neutralization with 15 μl 1 M Tris-HCl (pH 9.1), a 2 μl aliquot of the eluted phage suspension was taken for titering and the remainder was amplified in *E. coli* ER2738 for 4.5 h. After high-speed centrifugation, the supernatant was purified by precipitation with 20% PEG8000/2.5 M NaCl. Subsequently, the amplified phages were titered and used for the second biopanning.

Solution-phase panning by the affinity SPA Sepharose capture method was applied for second round biopanning as follows: the amplified phage was adjusted to 5 × 10^10^ pfu in TBST (Tween-20, 0.3%) and mixed with 0.5 μg antibodies. Following incubation for 20 min at ambient temperature, the phage-antibody mixture was added to 50 μl SPA Sepharose (Pierce, USA), and incubated for 15 min. Following washing 10x with TBST (Tween-20, 0.3%) and centrifugation, the adsorbed phages were eluted. Phages selected in this round were then titrated and amplified. The third round procedure was similar to the first, except that the Tween-20 concentration was adjusted to 0.5% (v/v).

### Selection of mimotopes by phage-ELISA

Phage plaques from the third round screening of each of the two phage libraries were randomly picked and amplified for ELISA as following: polyclonal antibodies were coated onto an ELISA plate (1 μg/well) and blocked with 0.5% BSA for 1 h. Purified phage clones, each containing 5 × 10^10^ phages, were added to the plate and incubated for 2 h at ambient temperature. After 6 washes with TBST (Tween-20, 0.5%), horseradish peroxidase (HRP)-conjugated anti-M13 monoclonal antibody (GE Healthcare, USA) was added and incubation was continued at ambient temperature for 1 h. Following an additional 6 washes with TBST, 50 μl TMB (Sigma, USA) was added, the plate was incubated for 10 min at 37°C, and then 50 μl 2 M sulfuric acid was added. OD_450_ was measured using a microplate reader (MK3; Thermo Labsystem, Finland). An absorbance value ≥2.1 × the mean value of a negative control (M13KE, vector phage without a displayed peptide) was considered positive.

### DNA sequencing and mimotope homology search against bacteria-encoded open reading frame (ORF)

After phage-ELISA detection, 100 positive phages were selected from each library for DNA sequencing (Genscript, Nanjing, China); the obtained sequences were analyzed as follows. First, mimotopes were searched in the SAROTUP database (http://immunet.cn/sarotup) to exclude target-unrelated peptides (TUPs; Huang et al., 2010). The Clustal Omega program (http://www.ebi.ac.uk/Tools/msa/clustalo/) was used for multiple-sequence alignment to identify core residues of the mimotopes. The peptides were then searched by BLASTP and PHI-BLAST in National Center for Biotechnology Information *E. rhusiopathiae*-encoded ORF database [Erysipelothrix (taxid:1647)]. Proteins with high similarity to each mimotope were selected for subcellular localization prediction.

### Surface proteins identification by subcellular localization prediction

The subcellular localization of proteins selected from the homology analysis of each mimotope was determined by searching the Cell-Ploc (http://www.csbio.sjtu.edu.cn/bioinf/Cell-PLoc), PSORTb (http://www.psort.org), and CELLO (http://cello.life.nctu.edu.tw) databases (Yu et al., 2006; Chou and Shen, 2010; Yu et al., 2010). Proteins that were identified as being located on the cell surface in at least two websites and that met the following conditions were selected as candidate surface antigens of *E. rhusiopathiae*: those showing high similarity to the mimotopes (≥ five continuous amino acid residues) or lower similarity to the mimotopes (≥ three but < five continuous amino acid residues; Vendruscolo et al., 2001) were evaluated for surface probability using Protean software, with corresponding amino acids showing the characteristics of functional epitopes determined based on the surface probability plot (DNASTAR, Madison, WI, USA).

### Cloning and expression of the selected surface proteins

Fourteen new proteins were defined and selected for cloning and expression in *E. coli*. The target sequence of the Fujisawa and SY1027 strains, available from GenBank (accession: AB019124 and CP005079), were used for primer design. The corresponding genes containing possible epitope regions (Table 1) were cloned in the pET-28a (+) vector (Novagen, Madison, WI, USA), and the recombinant proteins were expressed in *E. coli* BL21 (DE3). The oligonucleotide primers used to amplify the relevant DNA fragments from the template DNA of *E. rhusiopathiae* strain ML10.1 are listed in Table 2. Following expression in *E. coli*, soluble recombinant proteins were purified using a His-Bind Purification kit (Novagen) according to the manufacturer's instructions. Purity and concentration of the proteins were determined by SDS-PAGE and BCA Protein Assay Kit, respectively. Inclusion bodies were first dissolved in 8 M urea and purified by the same method as the proteins expressed in the supernatant. All recombinant proteins were used for the following immunization trial.

**Table 1:** **Homologous protein alignment analysis of mimotopes with ***E. rhusiopathiae*** in NCBI and subcellular localization prediction**.

**Mimotope**	**Possible epitope region**	**Sequencing proportion (%)**	**Possible surface protein**
^a^HINWPILPRLWV	^48^ PILPRI ^53^	12	Spa (Surface protective antigen, accession BAE93265, BAE93266, ALJ77816, ABR27208, etc.)
^a^SPDHASRDWRSR	^446^ SPDH ^449^	5	
^b^MSLLTAT	^14^ MSCLLLTA ^21^	3	
^b^DLKSTVK	^395^ DL*E*S*L*VK ^401^	2	
^a^TQLDDHKRSHHA	^865^ TQLD ^868^	10	RspA (rhusiopathiae surface protein A, accession BAK31725)
^a^ISRPTPSVNPLM	^1936^ RPTKPSVPPL ^1945^	1	
^a^LTQGTSDMTRHL	^651^ LTQ*Q*TS ^656^	1	
^b^NNWLTMP	^99^ TIKLTMP ^105^	2	
^b^NTVRESV	^1152^ VRESGV ^1157^	2	
^a^SWKPYTWKDTAL	^1347^ YTWKN ^1351^	1	RspC (rhusiopathiae surface protein C, accession BAK32739)
^a^FQGSPKTNHGKI	^1032^ SPKT ^1035^	1	
^a^GPTFNLQRIPAT	^1144^ TFNLYQI ^1150^	1	
^b^VSRYNWG	^1027^ VNRYQS ^1032^	1	
^b^SHNQPYQ	^786^ NQPY^789^	1	
^b^LQAKPRT	^458^ QAKP ^461^	1	
^a^QTKSSVYMMSYL	^258^ SVYL*Q*SY ^264^	5	CwpA (LPXTG-motif cell wall anchor domain protein, accession BAK32481)
^b^LSKWTTS	^948^ WTTS ^951^	2	
^b^LNANTSL	^226^ NANTAL^231^	2	
^b^SPNYSRN	^794^ SPNYVSKN ^801^	2	
^a^WHSNNSHNDSWP	^349^ NNTHDD^354^	1	Plp (Pectin lyase fold-containing protein, accession BAK31224)
^a^ADKPRVDTTTYN	^1465^ ADKP ^1468^	1	
^b^NADKPTE	^1465^ ADKPTD ^1469^	6	
^b^ERTNSSD	^421^ NSSD ^424^	1	
^b^QPSRDTY	^399^ SRDT ^402^	1	
^a^IRLPASLLLDPA	^1245^ P*T*SL*V*LD ^1251^	3	CbpA (Collagen-binding protein, accession BAK31134)
^b^YGDSNLA	^78^ DSNL^81^	2	
^b^GQEGMKE	^1325^ GQDGM ^1329^	2	
^a^GPKSNNVGVTYS	^746^ PKSN ^749^	4	Gh (Glycoside hydrolase, family 85, accession BAK31618)
^b^NAMLRAV	^237^ NAMLR ^241^	1	
^b^LPFMIHN	^965^ PFMIKN ^970^	1	
^a^FWPHKHNLYMST	^363^ PHKGNL ^368^	2	CbpB (Choline-binding protein, accession BAK31823)
^b^ILSDSGS	^113^ ILSDS ^117^	2	
^b^PTVKQKW	^511^ IKQKW ^515^	2	
^a^AHWFGGVYNKTM	^128^ FGGVY ^132^	1	Bga (Beta-galactosidase, accession BAK31835)
^a^TPLPSPIDITYQ	^1320^ PSLLDITYE ^1328^	1	
^a^YALHDGTAHNTW	^826^ AQHDGT ^831^	1	
^b^SSATLVY	^538^ SSAT^541^	1	
^a^LQASAKTMHGTI	^185^ LQEGAKSMNKTI^196^	3	Bml (Basic membrane lipoprotein, accession BAK32060)
^b^GAKWMSQ	^188^ GAKSM ^192^	2	
^a^SNTYLPKSYLNV	^433^ N*I*YL*K*KS^439^	1	Neu (Neuraminidase, accession BAK31357)
^b^RDKALVN	^698^ DKALI ^702^	1	
^b^NTTQSVM	^712^ NTTQ^715^	1	
^b^STSFDNS	^579^ TSYDN ^583^	1	
^a^AHRYIDAQIDRR	^191^ AQIDR ^195^	1	CwpB (LPXTG-motif cell wall anchor domain protein, accession BAK32507)
^b^LALDRRD	^191^ AQIDR ^195^	1	
^b^YRPTTDQ	^399^ YRPTTD ^401^	1	
^a^SMLRQEFPPTEP	^262^ DFPPT ^266^	1	Hp (alpha/beta hydrolase domain-containing protein, accession)
^b^LKTLNPS	^196^ TLNPS ^200^	1	
^a^QTHHHTFFMKSK	^274^ HHSNFMSNK ^282^	1	CbpC (Collagen-binding protein, accession BAK32489)
^b^QYRNHAD	^950^ RNQAD ^954^	1	
^a^YYYADDGAILLN	^556^ FYFADD ^561^	2	Da (Dipeptidyl aminopeptidase, accession BAK31118)
^b^SFPKNLD	^45^ PKNLD ^49^	1	Atsp (ABC transporter, substrate-binding protein, accession BAK31287)
^b^FQPKRLD	^45^ PKNLD ^49^	1	
^b^SSSGQRL	^1136^ SSGERL^1141^	1	Hya (Hyaluronidase, accession BAK31820)
^b^YRENHSY	^1293^ RQNHS ^1297^	1	
^b^ILNYPES	^931^ YPES ^934^	1	Pca (peptidase M14, carboxypeptidase A, accession BAK31220)
^b^QEGTNKA	^1012^ TNKA ^1015^	1	

a *12-peptides screened from random library Ph.D.™-12*.

b *7-peptides screened from random library Ph.D.-C7C; underlined sequences: may be the core residues and used for PHI-BLAST (performs the search but limits alignments to those core residues in the query); red font: may be the epitope region of the protein according to homologous protein alignment analysis of mimotope; red italic font: showing lower similarity to the mimotope but identified as a possible functional epitope by Protean software (surface probability analysis, DNAStar, Inc. Madison, WI)*.

**Table 2:** **PCR primers used for cloning of recombinant proteins**.

**Primer name**	**Primer sequence (5′–3′)**	**Target position**
CwpA Fp	CGCGGATCC GGGCTAGATGTTGATACTGATTTT	692–941aa
CwpA Rp	CCGCTCGAG ATTACCTGATGCATCAAATCTTTC	
Plp Fp	CGCGGATCC TTTTCCATCGATCGTGAGAATGG	862–1500aa
Plp Rp	ACGCGTCGAC TTCAAATTTTGATTGTACACCCG	
CbpA Fp	CGCGGATCC ACAACAGATGAAAAACTATCAGGT	26–350aa
CbpA Rp	CCGCTCGAG TGGTGCTTTGGTCTCATTCCAAC	
Bga Fp	CGCGGATCC TCATATAAAGCAATCCGTGCAAAAC	519–936aa
Bga Rp	CGCGTCGAC TACGCTAAATGATCCAGGTTGTTC	
BML Fp	GGAGAATTC ATGTCAAAGTCAACGGAAGATCAAAAG	20–362aa
BML Rp	TGTCGAC GCGATAATAATCGATTTTTAGAT	
Neu Fp	CGCGGATCC AAAGGGAATACACAAGTAGAAGGTC	837–1156aa
Neu Rp	CGCGTCGAC TGGATCTACGATTGGAGGTGTG	
CwpB Fp	CCCAAGCTT TGGGTTACTAAAGCACAAATTGAT	186–539aa
CwpB Rp	CCGCTCGAG TCCTTCAAACCACGCATCAACAC	
Da Fp	CGCGGATCC GCTAAGAAACACATGGCATTTATTAA	23–635aa
Da Rp	CGCGTCGAC AGTACGTGAAAGATCATGGTTTTC	
Atsp Fp	CGCGGATCC ACTGATAAACCAGAGGGAGAAG	26–481aa
Atsp Rp	CCGCTCGAG TTTACTTGCTTGGAATGCATCGA	
Hya Fp	CGCGGATCC ATGCCACATCAACGACTTGAATC	818–1509aa
Hya Rp	ACGCGTCGAC CATGTCTTGAGCACGACCTG	
Pca Fp	CGCGGATCC GGCGCAGAAGCAGATATCTTCC	602–1174aa
Pca Rp	CCGCTCGAG TTTTTTCTTTTTTGAGAACCAGTAG	

*Underlined sequences: restriction enzyme cutting sites. Recombinant proteins, Gh, CbpC, and Hp, were not successfully expressed and the corresponding primers are not listed. The CwpA, Plp, CbpA, Bga, and Neu genes code for multi-fragment expression, but only the fragment of each with the highest antigenicity is shown*.

### Immunogenicity of recombinant proteins

Female Institute of Cancer Research mice (3 weeks old) were immunized by subcutaneous injection with one of 11 recombinant proteins adsorbed to an aluminum-containing adjuvant (Brenntag Biosector, Fredrikssund, Denmark; 20 μg/mouse). After 2 weeks, the mice received a booster of the same recombinant protein with adjuvant. TBS and inactivated E. rhusiopathiae (5 × 10^7^ CFU/mouse) were used as negative and positive controls, respectively. Tail vein blood samples were collected before the first injection and every 14 days post injection, and serum antibody titers were determined by ELISA using recombinant proteins as antigens. Results were considered as positive if the OD_450_ of the positive relative to the negative control was >2.1. Two weeks after the boost immunization, the mice were challenged by intraperitoneal injection of 300 CFU of E. rhusiopathiae, and mortality was monitored for the following 14 days. The animal research was performed with the approval of the Review Committee for the Use of Animal Subjects of Hunan Agricultural University and all experimental protocols were performed according to the ARRIVE guidelines of the National Institutes of Health guide for the care and use of Laboratory animals (NIH Publications No. 8023, revised 1978). Survival profiles were plotted for each infected group and analyzed with the log-rank/Mantel-Cox test using GraphPad Prism 5 software (GraphPad Inc., La Jolla, CA, USA).

### Whole-cell ELISA and immunoblotting

Sera from mice immunized with the recombinant proteins were used to detect interactions with whole bacteria by whole-cell ELISA. A 96-well microtiter plate was coated with 10^7^ bacteria per well and adsorbed antibodies were detected as previously described (Pizza et al., 2000). Each assay was performed in duplicate. The surface exposure of the identified proteins was determined by immunoblotting, with swine polyclonal antibodies (prepared by affinity-purifying polyclonal antibodies against E. rhusiopathiae) against surface proteins of E. rhusiopathiae used as the primary antibody.

## Results

### Serum preparation and polyclonal antibody purification

**Figure 1:**
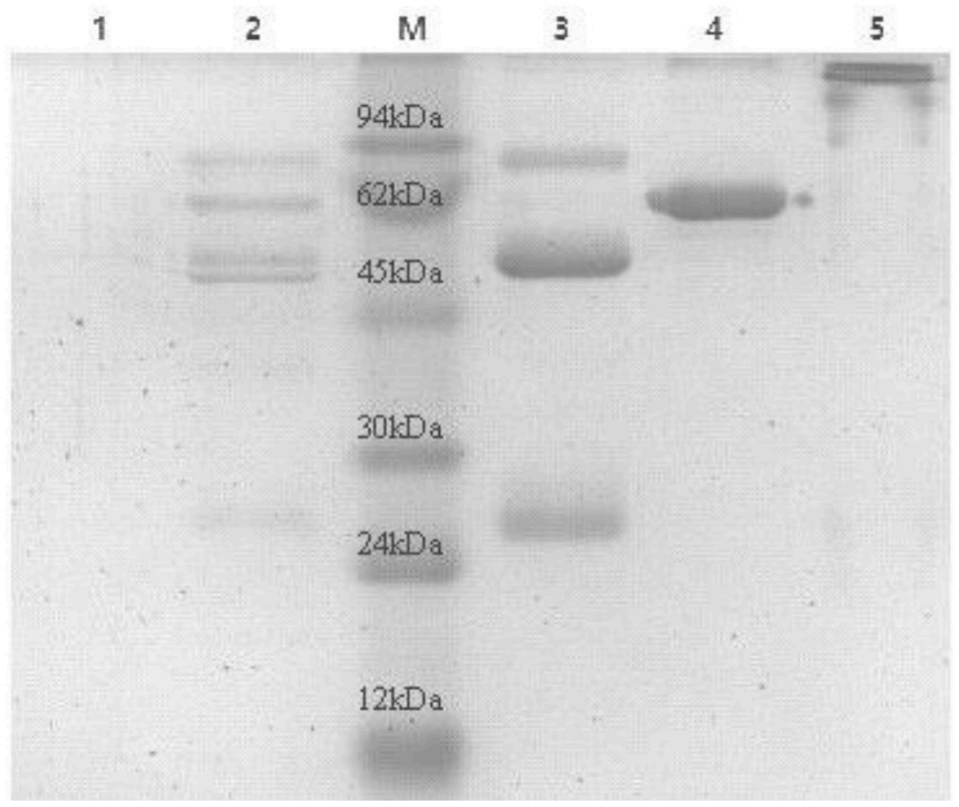
**SDS-PAGE analysis of ***E. rhusiopthiae*** polyclonal antibody with or without β-mercaptoethanol**. M, protein marker; 1, negative control; 2, polyclonal antibodies against *E. rhusiopthiae* before purified by recombinant Staphylococcus Protein A affinity chromatography; 3, polyclonal antibodies against *E. rhusiopthiae* in reductive (β-mercaptoethanol) SDS-PAGE after complete purification; 4, BSA (250 μg/ml); 5, polyclonal antibodies against *E. rhusiopthiae* in non-reductive SDS-PAGE after complete purification.

Eight antisera of E. rhusiopathiae derived from vaccine-immunized and bacterially challenged pigs were pooled and used for antibody purification. Highly pure polyclonal antibodies against E. rhusiopathiae surface molecules were obtained (antibody titers are shown in Supplementary Table 1; no antibodies were obtained from the negative control), as determined by SDS-PAGE (Figure 1). The concentration of the purified antibodies was 240 μg/ml, as determined by the BCA protein assay, indicating that the purification procedure was effective.

### Phage display and sequence analysis

Affinity purified polyclonal antibodies were used for phage display. After three rounds of biopanning, clones from each phage library were randomly selected and detected by phage-ELISA. Each phage library was selected 100 positive phages for DNA sequencing. A total of 36 separate 12-peptide and 65 separate 7-peptide mimotopes were obtained and no TUPs were found (data not shown). A bioinformatics analysis revealed 23 12-peptide and 34 7-peptide sequences showing high similarity to 18 surface proteins of *E. rhusiopathiae* (Table 1), of which 14 were targeted by both libraries. In particular, LQASAKTMHGTI and GAKWMSQ, ADKPRVDTTTYN and NADKPTE, and AHRYIDAQIDRR and LALDRRD showed high similarity in the same amino acid regions (i.e., possible epitopes) of Bml, Plp, and CwpB respectively (Table 1). Moreover, Spa, RspA, CbpB, and RspC—which are *E. rhusiopathiae* surface proteins (Shimoji et al., 2003; Ogawa et al., 2011; Shi et al., 2013; Uchiyama et al., 2014)—were also identified, although RspC is not a protective antigen.

### Cloning and expression of selected proteins and their immunogenicity analysis in mice

Of the 14 new proteins, CwpA, Plp, CbpA, Gh, Bga, Bml, Neu, CwpB, Hp, CbpC, Da, Atsp, Hya, and Pca, were selected and the corresponding genes were expressed in *E. coli* BL21 (DE3). Eleven proteins were obtained (SDS-PAGE see Supplementary Figure 1), including 8 inclusion bodies (Plp, CbpA, Bga, Neu, CwpB, Da, Hya, and Pca) and 3 soluble recombinant proteins (CwpA, Bml, and Atsp). However, three of the recombinant proteins (Gh, Hp, and CbpC) were not expressed in *E. coli*. The immunogenicity of the recombinants was evaluated by immunization; all of the mice developed high titers of antibody against each protein (see Supplementary Table 2).

**Figure 2:**
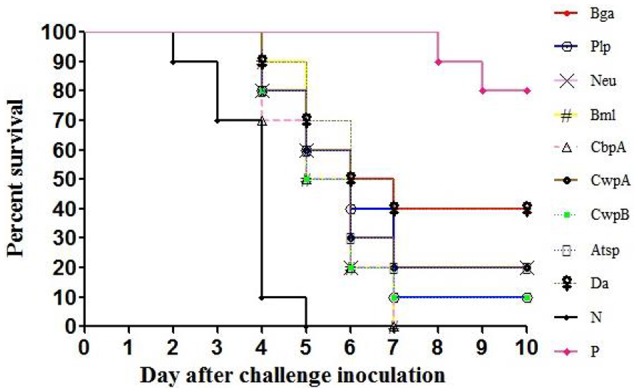
**Protection of mice against ***E. rhusiopathiae*** by the recombinant proteins**. N, negative control (TBS); P, positive control (inactivated vaccine of *E. rhusiopathiae*). Recombinant proteins, adsorbed to an aluminum-containing adjuvant, were used to immunize 3-week old female Institute of Cancer Research (ICR) mice by subcutaneous injection (20 μg/mouse). After 2 weeks, the mice received a booster of the same recombinant proteins with adjuvant. TBS and inactivated vaccine of *E. rhusiopathiae* (5 × 10^7^ CFU/mouse) were used as negative and positive controls, respectively. Two weeks after the last immunization, the mice were intraperitoneally challenged with 300 CFU of *E. rhusiopathiae*, and the mortalities were monitored for the following 14 days. Survival profiles were plotted for each infected group and analyzed with the log-rank/Mantel-Cox test using GraphPad Prism 5 software, *p* < 0.0001.

The LD_50_ of *E. rhusiopathiae* for mice was approximately 10 CFU; 300 CFU of bacteria were intraperitoneally injected into each mouse 14 days after boost immunization. In the Hya, Pca, and negative control groups, some mice developed clinical signs such as reduced activity, awkward movement, huddled posture, or ocular discharge and started to die on day 2 after the challenge. Some mice in the other groups—except for the positive controls—exhibited clinical symptoms on day 3 and started to die on day 4. Nine of the recombinant proteins provided protection against *E. rhusiopathiae* (*P* < 0.0001), with a survival rate of 10–20% among mice treated with CwpA, Neu, Atsp, Plp, or CwpB and a survival rate of 40% among those treated with Bga or Da (Figure 2). Mice in the Hya, Pca, and negative control groups all died by day 5, whereas those treated with Bml or CbpA died on day 7 after challenge; these deaths were significantly delayed relative to controls (*P* < 0.0001). Two of 10 mice in the inactivated vaccine group also died (see Supplementary Figures 2, 3, Supplementary Tables 3, 4).

### Surface-exposed analysis by whole-cell ELISA and immunoblotting

**Figure 3:**
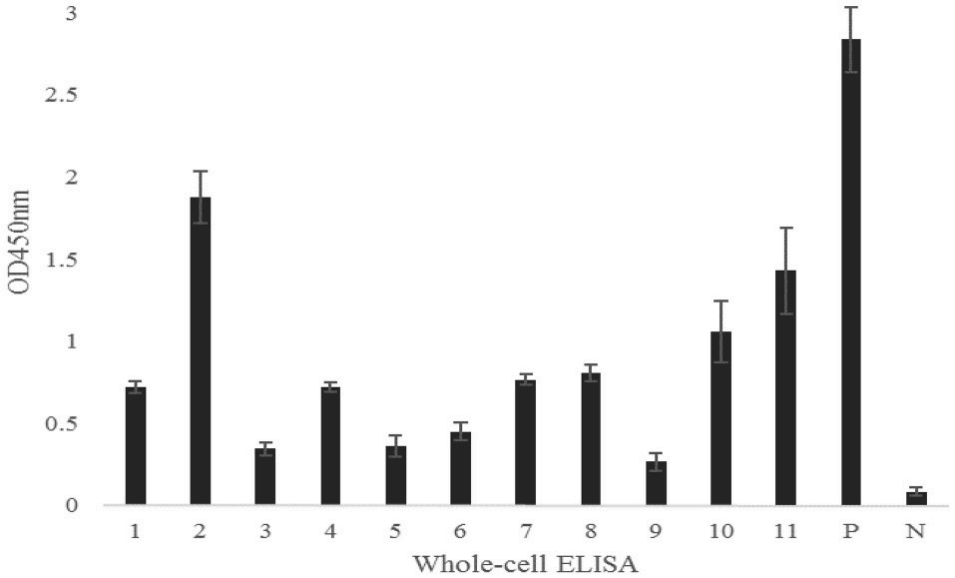
**Serum antibodies of recombinant proteins against ***E. rhusiopathiae*** as determined by whole-cell ELISA**. 1, Bga; 2, Hya; 3, Da; 4, Plp; 5, Bml; 6, Atsp; 7, Neu; 8, CbpA; 9, Pca; 10, CwpA; 11, CwpB; P, Positive control; N, Negative control. The error bars represent standard deviation of the mean from two independent experiments.

**Figure 4:**
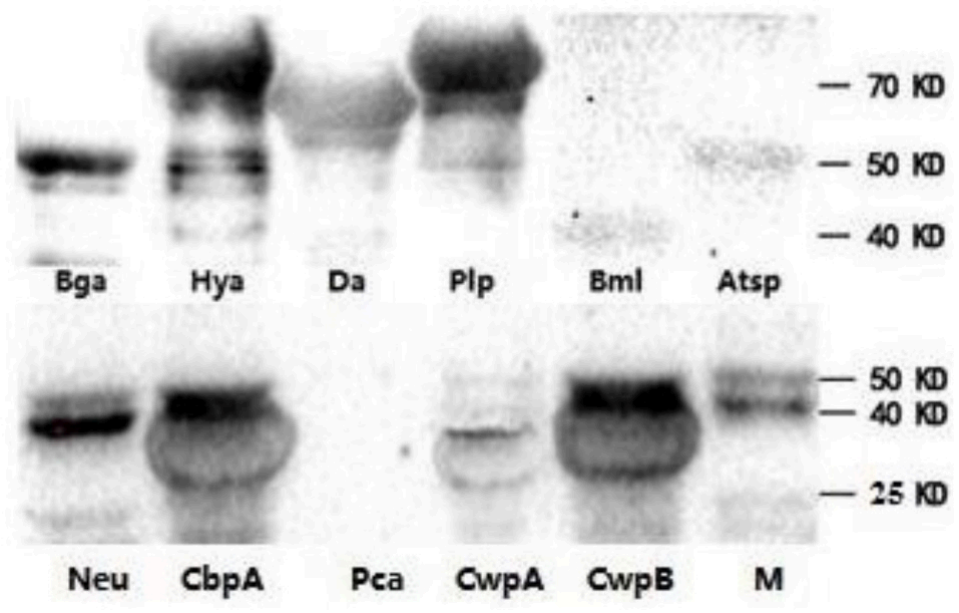
**Recombinant proteins of ***E. rhusiopathiae*** analyzed by western blots**. Purified swine IgG against surface molecules of *E. rhusiopathiae* was used as the primary antibody. M, protein marker; three images are grouped together.

To determine if the identified proteins are *E. rhusiopathiae* surface proteins, sera against each recombinant were tested by whole-cell ELISA. All serum antibodies against the recombinants were positive by ELISA, with anti-Hya and -CwpB antibodies present at high OD_450_ values, and anti-Pca, -Bml, -Atsp, and -Da antibodies present at low OD_450_ (Figure 3). To evaluate the surface exposure of each protein *in vivo*, we performed immunoblotting of the 11 recombinant proteins using swine polyclonal antibodies against *E. rhusiopathiae* surface molecules. The results showed that apart from Pca, Bml and Atsp all recombinant proteins stimulated a strong immunogenic reaction (Figure 4, full-length gels and blots can be found as Supplementary Figure 3).

## Discussion

To obtain polyclonal antibodies against only the surface molecules of bacteria, antisera were mixed with whole *E. rhusiopathiae* cells to allow the adsorption of target antibodies onto the bacterial surface; the antibodies were then affinity purified (Figure 1). A key aspect of the new approach reported here is the high efficiency with which surface proteins can be identified according to bacterial mimotopes. In theory, mimotopes screened using whole bacterial cell-purified antisera can be used to identify the corresponding antigen genes through a homology search of the bacterial genome. However, this is challenging in practice since hundreds of proteins can be identified by homology analysis (BLAST search) of the short mimotope input sequences. For instance, when the 12-peptide mimotope HINWPILPRLWV was searched against the full *E. rhusiopathiae* genome, more than 70 homologous proteins were listed. However, by applying the subcellular localization criterion, surface proteins can be specifically detected.

Solid- and solution-phase panning procedures as well as linear and conformational phage libraries were alternately applied to detect appropriate mimotopes. About 64% (23/36) of 12-peptides and 53% (34/65) of 7-peptides are linked to a surface protein, with 14/18 found by both phage libraries. In particular, LQASAKTMHGTI, GAKWMSQ, ADKPRVDTTTYN, NADKPTE, AHRYIDAQIDRR, and LALDRRD showed high similarity to the same amino acid regions within Bml, Plp, and CwpB (Table 1), indicating that our biopanning approach was effective. However, it should be noted that mimotopes cannot be used to find all native surface proteins of *E. rhusiopathiae*, since some may mimic polysaccharides and lipids that are also detected by affinity selection (Neu, 1996; Srivastava et al., 2000).

Of a total of 18 surface proteins screened, Spa—which is a major protective antigen of *E. rhusiopathiae*—had the highest proportion of screening hits (17% for 12-peptides and 5% for 7-peptides; Table 1). Other reported surface protective antigens including RspA and CbpB (Shimoji et al., 2003; Shi et al., 2013; Uchiyama et al., 2014)—which were detected by both libraries—also showed a high proportion of hits. RspC (with a proportion of screening hits of 3% in both libraries) was also identified as a surface protein of *E. rhusiopathiae*, although not as a protective antigen (Ogawa et al., 2011). In addition, nine new protective antigens with 7 were identified by both phage libraries. These results demonstrate that this is a highly effective procedure for protective antigen screening.

Reverse vaccinology and proteomics technologies have been used to identify protective bacterial antigens. The former was first applied to the identification of seven meningococcal surface protective antigens from among 350 candidates during an 18-month period (Pizza et al., 2000). Protective antigens have also been screened from a variety of surface proteins using a cell wall proteomics approach. For example, six protective antigens were identified from among 114 surface proteins of *S. pneumoniae* (Olaya-Abril et al., 2012), and 14 were identified from among 75 surface proteins of group A *Streptococcus*, but with a low screening efficiency (< 20%; Rodríguez-Ortega et al., 2006). The efficiency can be improved by immunoproteomics; nonetheless, cell wall separation and pretreatment steps required in these procedures are labor intensive (Emerson et al., 2008). In our approach, three previously reported and nine new protective antigens were identified from among 11 proteins within a 6-month period, with a screening efficiency >80%. In addition, our approach is uncomplicated and requires no specialized instrumentation, thus saving time and cost.

We evaluated immunogenicity and surface exposure of selected proteins in the test bacterium. We found that Hya is a highly expressed surface-exposed protein in *E. rhusiopathiae* (Figures 3, 4), but is not a protective antigen for reasons that are unclear. Of the nine identified protective antigens, the discovery of Bga and Da is surprising, since both are thought to be involved in metabolic regulation. Bga plays an important role in lactose metabolism and is released by bacteria, although *S. pneumonia* Bga may function in pathogenesis rather than in lactose enzymolysis (Zähner and Hakenbeck, 2000). In our study, *E. rhusiopathiae* Bga protected 40% of mice against infection; however, given its high similarity to *S. pneumonia* Bga it may also be involved in *E. rhusiopathiae* pathogenesis.

Aminopeptidases are involved in the synthesis and degradation of physiological components of bacteria, including in post-translational modification, signal peptide cutting, and proenzyme activation (Jankiewicz and Bielawski, 2003). Our results revealed that the aminopeptidase Da conferred protection to 40% of mice against bacterial challenge and may present a higher surface-exposed *in vivo* relative to *in vitro* (Figures 3, 4), suggesting that the antibody induced by Da plays an important role in immune protection against *E. rhusiopathiae*. These findings suggest that our approach can provide novel insights in functional studies of bacterial surface protective antigens.

We have described a new approach for identifying bacterial protective antigens by screening peptide phage libraries using whole bacterial cell-purified antisera with an efficiency of up to 80%. This enables the rapid discovery of protective antigens with manageable amounts of biopanning and bioinformatics analysis while providing insights into the functions of bacterial surface antigens. Protective antigens of strain D *P. multocida* (a Gram-negative species) were also screened in this manner, and the results confirmed the high efficiency of this method (unpublished work in our laboratory). Application of this approach to other pathogens may promote the discovery and improve functional analyses of candidate surface proteins against infectious diseases.

## Author contributions

YFH and XY designed the experiment; YFH performed the antibody purification and phage display experiments; YFH and DZ performed the bioinformatics analysis; YFH, YLH, TX, XL, and HL performed cloning, expression, and immunogenicity experiments; YFH, XY, RL, and MG analyzed the data; YFH and XY wrote the manuscript with input from all authors who reviewed the final paper.

## Funding

This work was supported by the National Natural Science Foundation of China (NO. 31672539).

### Conflict of interest statement

The authors declare that the research was conducted in the absence of any commercial or financial relationships that could be construed as a potential conflict of interest.
