# Supplementary material for: Identification of Bacterial Surface Antigens by Screening Peptide Phage Libraries Using Whole Bacteria Cell-Purified Antisera
Source: Front Microbiol. 2017 Jan 26;8:82. doi: 10.3389/fmicb.2017.00082 (PMC5266700; doi:10.3389/fmicb.2017.00082)
Supplement: Supplementary file 1 [file Table2.DOCX]

***Supplementary Material***

**Efficient discovery of bacterial surface protective antigens by using a combined approach of phage display and subcellular localization**

Yun-Fei Hu, Dun Zhao, Xing-Long Yu*, Yu-Li Hu, Run-Cheng Li, Meng Ge, Tian-Qi Xu, Xiao-Bo Liu, Hua-Yuan Liao

* **Correspondence:** Corresponding author: xlyu999@126.com

**1 Supplementary Figures and Tables**

**1.1 Supplementary Figures**


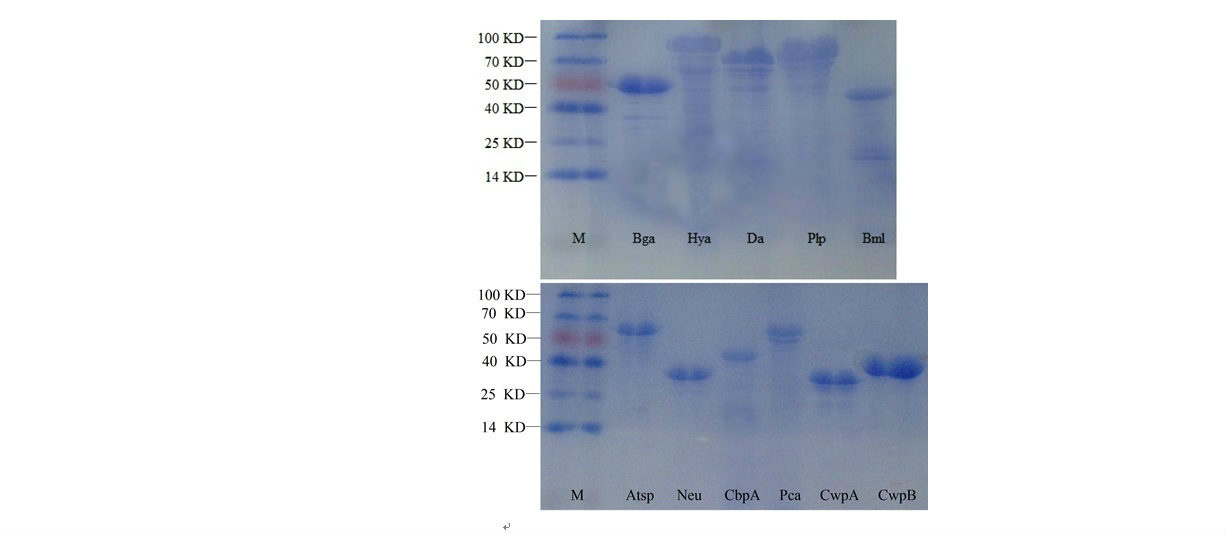


**Supplementary Figure S1.** SDS-PAGE of the 11 recombinant proteins of *E. rhusiopathiae*. M: protein marker.

Recombinant proteins, adsorbed to an aluminum-containing adjuvant, were used to immunize 3-week old female Institute of Cancer Research (ICR) mice by subcutaneous injection (20 μg/mouse). After two weeks, the mice received a booster of the same recombinant proteins with adjuvant. TBS and inactivated vaccine of *E. rhusiopathiae* (5×10^7^ CFU/mouse) were used as negative and positive controls, respectively. Two weeks after the last immunization, the mice were intraperitoneally challenged with 300 CFU (approximately 30x the 50% lethal dose [LD_50_]) of *E. rhusiopathiae*, and the mortalities were monitored for the following 14 days. N: negative control, P: positive control. The survival profiles for each infected group were plotted and analyzed by using GraphPad Prism 5 software (log-rank (Mantel-Cox) test), p<0.0001.

**Supplementary Figure S2**. Survival curve of mice against *E. rhusiopathiae* after immunized with the recombinant proteins. N: negative control (TBS); P: positive control (inactivated vaccine of *E. rhusiopathiae*).


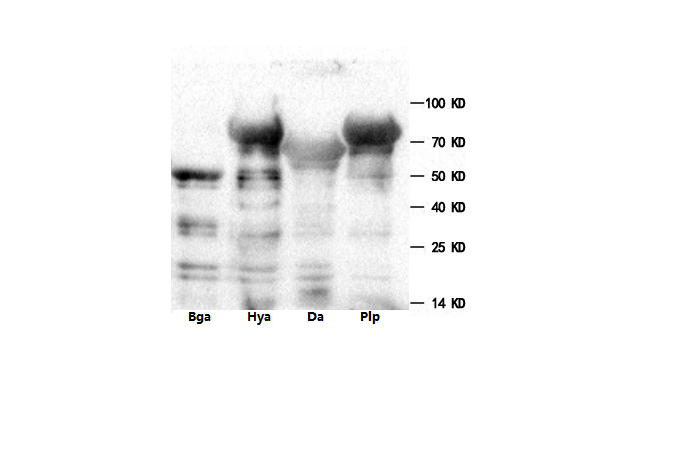

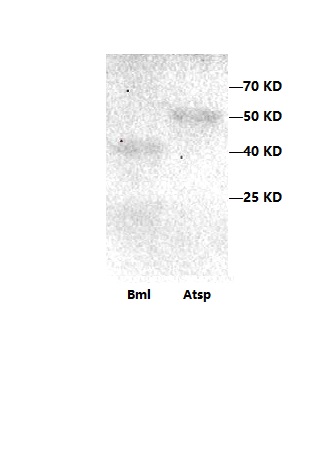


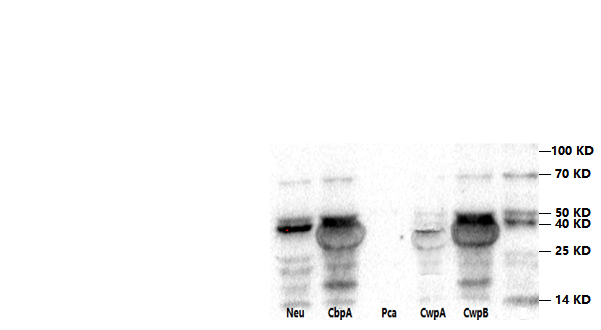


**Supplementary Figure S3.** Recombinant proteins of *E. rhusiopathiae* analyzed by western blots. Purified swine IgG against surface molecules of *E. rhusiopathiae* was used as the primary antibody.

**1.2 Supplementary Tables**

**Supplementary Table S1.** Titer determination of the purified polyclonal antibodies against *E. rhusiopathiae*

| Titer | OD*_450_* | | |
| --- | --- | --- | --- |
|  | pooled antisera | purified polyclonal antibodies | negative control |
| 1∶100 | 2.087 | 2.220 | 0.155 |
| 1∶200 | 1.796 | 1.921 | 0.167 |
| 1∶400 | 1.415 | 1.687 | 0.123 |
| 1∶800 | 0.990 | 1.489 | 0.075 |
| 1∶1 600 | 0.677 | 1.248 | 0.021 |
| 1∶3 200 | 0.445 | 0.867 | 0.081 |
| 1∶6 400 | 0.302 | 0.613 | 0.064 |
| 1∶12 800 | 0.210 | 0.425 | 0.056 |

**Supplementary Table S2.** Titer determination of the antibodies against recombinant proteins

| Protein | Antibody titer | Protein | Antibody titer | Protein | Antibody titer |
| --- | --- | --- | --- | --- | --- |
| Bga | 1:25600 | Bml | 1:12800 | Pca | 1:12800 |
| Hya | 1:25600 | Atsp | 1:25600 | CwpA | 1:25600 |
| Da | 1:25600 | Neu | 1:12800 | CwpB | 1:12800 |
| Plp | 1:12800 | CbpA | 1:25600 |  |  |

**Supplementary Table S3.** Immunogenicity of the 11 recombinant proteins (1)

| Day after challenge inoculation | The numbers of survival mice | | | | | | |
| --- | --- | --- | --- | --- | --- | --- | --- |
|  | Bga | Plp | Neu | Bml | Hya | N | P |
| 1 | 10 | 10 | 10 | 10 | 10 | 10 | 10 |
| 2 | 10 | 10 | 10 | 10 | 10 | 10 | 10 |
| 3 | 10 | 10 | 10 | 10 | 8 | 8 | 10 |
| 4 | 8 | 8 | 8 | 9 | 3 | 3 | 10 |
| 5 | 6 | 6 | 6 | 5 | 0 | 0 | 10 |
| 6 | 5 | 4 | 2 | 2 | 0 | 0 | 10 |
| 7 | 4 | 1 | 2 | 0 | 0 | 0 | 10 |
| 8 | 4 | 1 | 2 | 0 | 0 | 0 | 10 |
| 9 | 4 | 1 | 2 | 0 | 0 | 0 | 9 |
| 10 | 4 | 1 | 2 | 0 | 0 | 0 | 9 |
| 11 | 4 | 1 | 2 | 0 | 0 | 0 | 9 |
| 12 | 4 | 1 | 2 | 0 | 0 | 0 | 9 |
| 13 | 4 | 1 | 2 | 0 | 0 | 0 | 9 |
| 14 | 4 | 1 | 2 | 0 | 0 | 0 | 9 |

N: negative control, P: positive control.

**Supplementary Table S4.** Immunogenicity of the 11 recombinant proteins (2)

| Day after challenge inoculation | The numbers of survival mice | | | | | | | |
| --- | --- | --- | --- | --- | --- | --- | --- | --- |
|  | CbpA | CwpA | CwpB | Atsp | Da | Pca | N | P |
| 1 | 10 | 10 | 10 | 10 | 10 | 10 | 10 | 10 |
| 2 | 10 | 10 | 10 | 10 | 10 | 10 | 10 | 10 |
| 3 | 10 | 10 | 10 | 10 | 10 | 6 | 8 | 10 |
| 4 | 7 | 8 | 8 | 8 | 9 | 2 | 2 | 10 |
| 5 | 5 | 6 | 5 | 6 | 8 | 0 | 0 | 10 |
| 6 | 2 | 3 | 2 | 3 | 5 | 0 | 0 | 10 |
| 7 | 0 | 2 | 1 | 2 | 5 | 0 | 0 | 10 |
| 8 | 0 | 2 | 1 | 2 | 4 | 0 | 0 | 9 |
| 9 | 0 | 2 | 1 | 2 | 4 | 0 | 0 | 8 |
| 10 | 0 | 2 | 1 | 2 | 4 | 0 | 0 | 8 |
| 11 | 0 | 2 | 1 | 2 | 4 | 0 | 0 | 8 |
| 12 | 0 | 2 | 1 | 2 | 4 | 0 | 0 | 8 |
| 13 | 0 | 2 | 1 | 2 | 4 | 0 | 0 | 8 |
| 14 | 0 | 2 | 1 | 2 | 4 | 0 | 0 | 8 |

N: negative control, P: positive control.
